# Supplementary material for: Fluctuations of the transcription factor ATML1 generate the pattern of giant cells in the Arabidopsis sepal
Source: eLife. 2017 Feb 1;6:e19131. doi: 10.7554/eLife.19131 (PMC5333958; doi:10.7554/eLife.19131)
Supplement: Supplementary file 1. — See readme files within the different folders for further information. All raw image confocal tif files and example image processing files may be downloaded from: http://dx.doi.org/10.7946/P29G6M DOI: http://dx.doi.org/10.7554/eLife.19131.046 [file elife-19131-supp1.zip › lineage files/Read_me.pdf]

This file includes both raw and selected lineage excel files (.xlsx).

Raw files include:

Lineage identification number = the BOA identification number of that nucleus. Lost tracking is reported as 1 or -1.
Concentration = concentration of fluorescent fusion protein (total fluorescent intensity normalized by area)
Area = area of the nucleus taken from the slice containing the largest cross section of the nucleus.
Eccentricity = eccentricity of the nucleus taken from the slice containing the largest cross section of the nucleus.

Raw files include all the nuclei detected in each flower, regardless of their tracking. Anything that says NaN lost the tracking.

Selected lineage files include:

All trackable lineages with high quality segmentation. A single info sheet contains the lineage, area, concentration, and determined ploidy.

The color scheme shows the ploidy: yellow = 2C, blue = 4C, and pink/red = 8C and higher.

The normalized sheet contains the mean normalized concentrations.
