# Supplementary figures and images for: Fluctuations of the transcription factor ATML1 generate the pattern of giant cells in the Arabidopsis sepal
Source: eLife. 2017 Feb 1;6:e19131. doi: 10.7554/eLife.19131 (PMC5333958; doi:10.7554/eLife.19131)

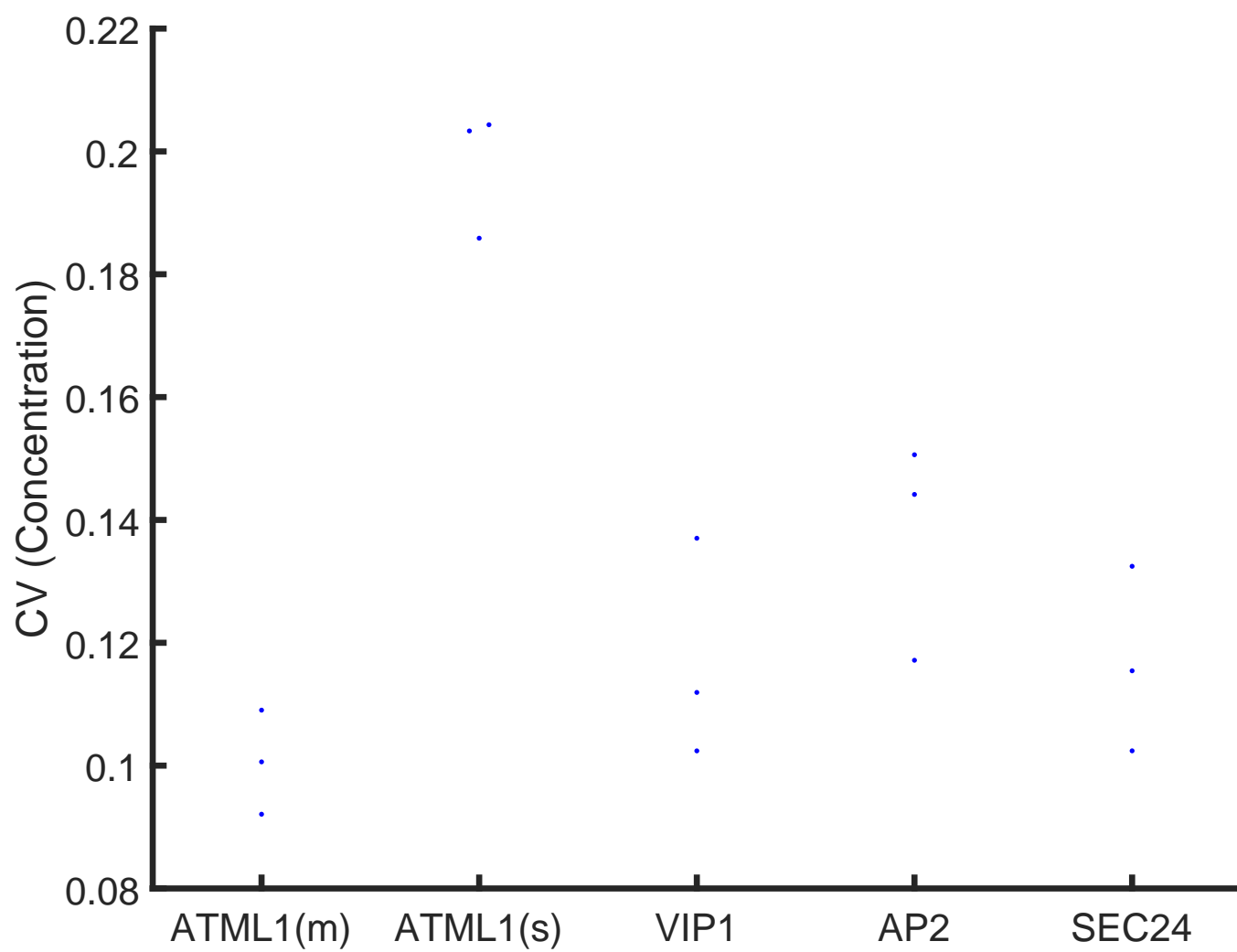

Supplement: Source code 1. — DOI: http://dx.doi.org/10.7554/eLife.19131.047 [file elife-19131-code1.zip › ATML1_Source_code_1/classComparison_CV.pdf]
